# Supplementary material for: Influenza A Virus Infection Reactivates Human Endogenous Retroviruses Associated with Modulation of Antiviral Immunity
Source: Viruses. 2022 Jul 21;14(7):1591. doi: 10.3390/v14071591 (PMC9320126; doi:10.3390/v14071591)
Supplement: Supplementary file 1 [file viruses-14-01591-s001.zip › MotifAnalysis.zip!/MotifOutput_PR8M/homerResults/motif30.similar.html]

motif30

## Information for motif30

A
C
T
G
A
G
T
C
C
G
T
A
A
G
T
C
A
G
T
C
A
G
T
C
A
C
G
T
A
C
T
G
  
Reverse Opposite:  

A
G
T
C
C
G
T
A
A
C
T
G
C
T
A
G
A
C
T
G
A
C
G
T
A
C
T
G
A
G
T
C
  

|  |  |
| --- | --- |
| p-value: | 1e-7 |
| log p-value: | -1.623e+01 |
| Information Content per bp: | 1.946 |
| Number of Target Sequences with motif | 6.0 |
| Percentage of Target Sequences with motif | 26.09% |
| Number of Background Sequences with motif | 460.0 |
| Percentage of Background Sequences with motif | 1.00% |
| Average Position of motif in Targets | 113.4 +/- 51.8bp |
| Average Position of motif in Background | 99.2 +/- 73.0bp |
| Strand Bias (log2 ratio + to - strand density) | 10.0 |
| Multiplicity (# of sites on avg that occur together) | 1.17 |
| Motif File: | file (matrix) reverse opposite |

### Similar de novo motifs found

|  |  |  |  |  |  |  |  |
| --- | --- | --- | --- | --- | --- | --- | --- |
| Rank | Match Score | Redundant Motif | P-value | log P-value | % of Targets | % of Background | Motif file |
